# Influence of Environmental Factors on the Paralytic Shellfish Toxin Content and Profile of *Alexandrium catenella* (Dinophyceae) Isolated from the Mediterranean Sea

**DOI:** 10.3390/md11051583

**Published:** 2013-05-15

**Authors:** Mohamed Laabir, Yves Collos, Estelle Masseret, Daniel Grzebyk, Eric Abadie, Véronique Savart, Manoella Sibat, Zouher Amzil

**Affiliations:** 1 Université Montpellier 2, Centre National de la Recherche Scientifique, Université Montpellier 1, IFREMER, IRD, UMR 5119 “Ecologie des Systèmes Marins Côtiers”, Place E. Bataillon, CC093, 34095 Montpellier cedex 5, France; E-Mails: yves.collos@univ-montp2.fr (Y.C.); estelle.masseret@univ-montp2.fr (E.M.); daniel.grzebyk@univ-montp2.fr (D.G.); 2 Laboratoire Environnement Ressources Languedoc-Roussillon Ifremer, B.P. 171, 34203 Sète, France; E-Mail: eric.abadie@ifremer.fr; 3 IFREMER, Phycotoxins Laboratory, Rue de l’Ile d’Yeu BP 21105 44311 Nantes CEDEX 3, France; E-Mails: veronique.savar@ifremer.fr (V.S.); manoella.sibat@ifremer.fr (M.S.); zouher.amzil@ifremer.fr (Z.A.)

**Keywords:** *Alexandrium catenella*, PSP toxins, temperature, salinity, light

## Abstract

Laboratory experiments were designed to study the toxin content and profile of the *Alexandrium catenella* strain ACT03 (isolated from Thau Lagoon, French Mediterranean) in response to abiotic environmental factors under nutrient-replete conditions. This dinoflagellate can produce various paralytic shellfish toxins with concentrations ranging from 2.9 to 50.3 fmol/cell. The toxin profile was characterized by carbamate toxins (GTX3, GTX4 and GTX5) and *N*-sulfocarbamoyl toxins (C1, C2, C3 and C4). C2 dominated at 12–18 °C, but only for salinities ranging from 10 to 25 psu, whereas GTX5 became dominant at temperatures ranging from 21 to 30 °C at almost all salinities. There was no significant variation in the cellular toxin amount from 18 °C to 27 °C for salinities ranging between 30 and 40 psu. At salinities of 10 to 25 psu, the toxin concentrations always remained below 20 fmol/cell. Toxin content was stable for irradiance ranging from 10 to 70 μmol photons/m^2^/s then slightly increased. Overall, the toxin profile was more stable than the toxin content (fmol/cell), except for temperature and/or salinity values different from those recorded during *Alexandrium* blooms in Thau Lagoon.

## 1. Introduction

**Figure 1:**
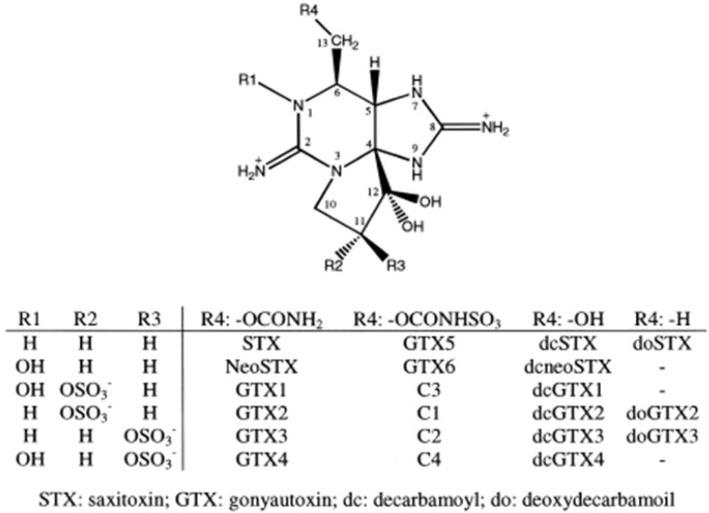
Common structure of paralytic shellfish toxins.

Many species in the genus *Alexandrium* are among the most harmful algal blooms (HAB) organisms [1,2]. *Alexandrium* blooms occur under a wide range of latitudes in coastal ecosystems that are characterized by different temperature, salinity, and light conditions [2,3]. *Alexandrium* species, as well as *Gymnodinium catenatum* and *Pyrodinium bahamense*, are often responsible for the production of paralytic shellfish poisoning toxins (PST) that can impact human health. PSP is a worldwide marine toxin syndrome with both neurologic and gastrointestinal symptoms resulting from the consumption of shellfish contaminated by toxic dinoflagellates. Saxitoxin and its congeners can be divided into three categories: the carbamate compounds, which include saxitoxin, neo-saxitoxin and gonyautoxins 1–4; the *N*-sulfocarbamoyl compounds, which include the C and B toxins; and the decarbamoyl compounds (Figure 1). These compounds vary in toxic potency by orders of magnitude due to certain minor structural differences [4]. Numerous studies reported that the toxin content (fmol/cell) and, to a lesser extent, the toxin composition (mol%) could vary with salinity [5,6,7,8], temperature [8,9,10], light intensity [8,11] and nutrients [12,13,14,15]. When considering the effect of nutrients on the toxin production, results showed that nitrogen depletion induces a decrease in toxin content of *Alexandrium* species [10,16], whereas P-limited growth induces a significant increase in the cell toxin content [10,16]. It has been hypothesized that the P-stressed cells allocate nitrogen to STX production instead of proteins synthesis [10,17]. Nevertheless, some authors have argued that the toxin composition could be considered as a stable biochemical characteristic and could be used as a genetic fingerprint for chemotaxonomic characterization of cultured strains [9,16,17,18,19].

Blooms of *Alexandrium catenella* increase in frequency and importance in many marine coastal areas around the world [2]. The expansion of this toxic species has been documented in the Mediterranean Sea in the last decade [20] with reports of extensive blooms in several coastal areas [21,22,23,24]. The Thau Lagoon has experienced recurrent blooms of the neurotoxic *A. catenella* during spring and autumn, reaching high cell concentrations (3 to 14 × 10^6^ cells/L), with toxin contamination in bivalves frequently exceeding the sanitary threshold [25,26]. In Thau, the rain pattern is characterized by strong inter-annual variability (200–1000 mm/year). The seasonal weather fluctuations impose a wide range of water temperatures (3–29 °C) and salinities (27–40 psu) [27]. Experimental data on environmental factors driving the PSP toxin content in dinoflagellates from various geographic regions and particularly from the Mediterranean Sea are still scarce. Laboratory experiments were designed to study the cell PST content of an *A. catenella* strain, ACT03, isolated from Thau Lagoon (Mediterranean Sea) in response to varying abiotic environmental factors. The present study examines for the first time the influence of irradiance and temperature/salinity on the paralytic shellfish toxin content of this organism grown in an artificial seawater medium under nutrient-replete conditions.

## 2. Results and Discussion

### 2.1. Toxin Content and Profile of ACT03 Strain

The paralytic shellfish toxins were determined by Liquid Chromatography/Fluorescence Detection (LC/FD) allowing to separate carbamoyl toxins (STX, NeoSTX, GTX1, GTX2, GTX3 & GTX4), *N*-sulfocarbamoyl toxins (C1, C2, C3, C4, B1 = GTX5 & B2 = GTX6) and decarbamoyl toxins (dc-STX, dc-GTX2 & dc-GTX3), the structure of these toxins is reported in Figure 1. Toxin profiles produced by *Alexandrium* species from different marine systems across many regions of the world are quite diverse [2,28]. However, data concerning PSP toxin profiles of *Alexandrium* in Mediterranean waters remain scarce [21,29,30,31,32,33] and this study represents the first detailed laboratory investigation on the variation in PSP toxin profiles in relation to changing environmental conditions for *A. catenella*.

**Figure 2:**
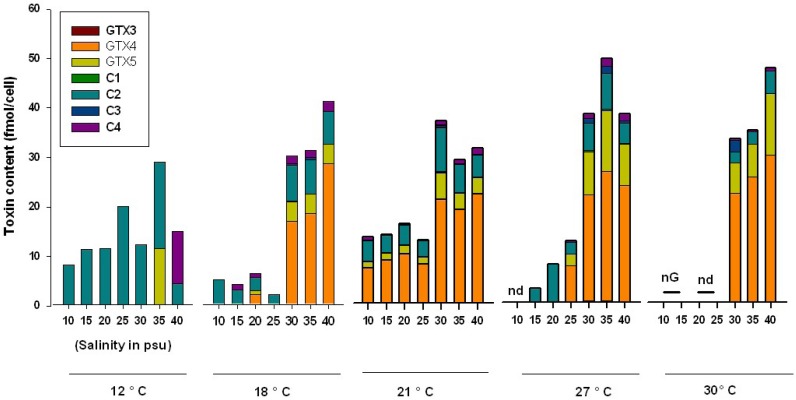
Toxin content (fmol/cell) and toxin profile of *Alexandrium catenella* grown at various combinations of temperature and salinity conditions. nd: toxins were not detected in the examined cells; nG: since cells did not grow, toxin content could not be analyzed.

The toxin profile of ACT03 cells from a culture harvested in late exponential growth phase included the toxins GTX3, GTX4, GTX5, C1, C2, C3 and C4 (Figure 2). Other scrutinized toxins including STX, NeoSTX or dc-STX were not detected. C2, C4, GTX4 and GTX5 were the major toxins produced, accounting for about 90 mol% toxin cell under the conditions of this study (Table 1).

Two strains of *A. catenella* isolated in 1998 from Thau Lagoon (ATTL01 and ATTL02) and grown in f/2 medium (salinity of 31 psu) at 15 °C and 200 μmol photons/m^2^/s showed in their exponential growth phase a dominance of C1,2 and GTX1,4,5, a profile nearly similar to that displayed by several Japanese *A. catenella* strains [21]. Significant regional variations in toxin profile were observed among *Alexandrium* populations. For example, *A. catenella* from China Sea showed a dominance of C1,2 toxins, whereas GTX 1 to 4 were minor (<15 mol%) or present as traces [34,35]. The GTXs dominated in *A. catenella* strains from Hong Kong waters with GTX4 > GTX3 > GTX1 > GTX6 [36]. In contrast, *A. catenella* from the southern coast of Chile showed a dominance of C1,2 and GTX1,4 [37] or the dominance of GTX 1 to 4, depending on the isolate. 

**Table 1:** Molar percentage (mol%) of the toxins in *Alexandrium catenella* (strain ACT03) cells grown at different temperature and salinity conditions.

Temperature (°C)	Salinity (psu)	GTX3	GTX4	GTX5	C1	C2	C3	C4
12	10	-	-	-	-	100	-	-
12	15	-	-	-	-	100	-	-
12	20	-	-	-	-	100	-	-
12	25	-	-	-	-	100	-	-
12	30	-	-	-	-	100	-	-
12	35	-	-	31	-	69	-	-
12	40	-	-	-	-	57.3	-	42.7
18	10	-	-	-	-	100	-	-
18	15	-	-	-	-	80.8	-	19.2
18	20	-	18.5	24.1	-	47.8	-	9.6
18	25	-	-	-	-	100	-	-
18	30	0.1	13.4	55.8	0.3	24	1.1	5.2
18	35	-	12.8	57.8	0.3	22.9	1.2	4.9
18	40	-	10.2	66.7	-	17.6	-	5.5
21	10	-	9.4	51.6	-	33.3	-	5.7
21	15	-	10.5	59.5	-	27.6	-	2.4
21	20	-	11	58.7	-	28.1	-	2.3
21	25	-	11.5	61.3	-	26.1	-	1.1
21	30	0.4	14.6	56.1	0.3	24.5	1.4	2.8
21	35	-	12.1	62.3	-	21.7	-	3.9
21	40	-	11.3	66.8	-	16.3	-	5.6
27	15	-	-	-	-	100	-	-
27	20	-	-	-	-	100	-	-
27	25	-	18.9	59	-	18.8	-	3.3
27	30	0.6	23.7	54.6	0.5	15.6	2.7	2.2
27	35	0.5	25.2	52.4	0.6	15.3	2.8	3.3
27	40	-	23.2	60	0.2	11.4	1	4.2
30	30	-	19.7	66.8	-	6.7	5.6	1.1
30	35	-	35	71.8	-	7.3	-	1.1
30	40	-	40	60.6	-	10.4	-	1.9

### 2.2. Influence of Salinity

**Figure 3:**
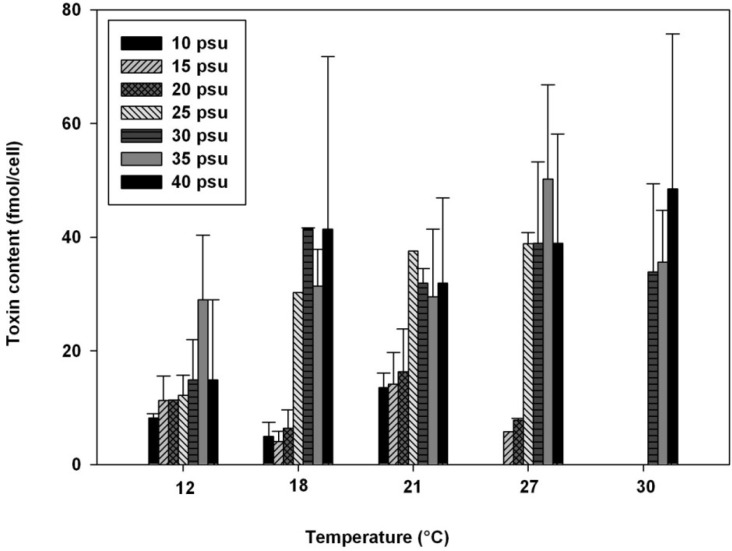
Total toxin content (fmol/cell) of *Alexandrium catenella* grown at various combinations of temperature and salinity.

In a previous work [38], ACT03 was shown to be a euryhaline strain that can survive at salinities as low as 10 psu and can grow under salinities up to 40 psu, showing optimal growth between 30 and 40 psu. It has been suggested that this organism is well adapted to the salinity conditions recorded for the Thau lagoon, usually ranging from 35 psu in winter to 39 psu in summer. Overall, at temperatures ranging between 12 and 30 °C, increasing the medium salinity from 10 to 40 psu caused an increase in the whole toxin content up to a maximum of 48.5 ± 27.3 fmol/cell and 50.3 ± 16.6 fmol/cell for two temperature/salinity combinations of 30 °C/40 psu and 27 °C/35 psu, respectively (Figure 3). For salinities lower than 25 psu, the toxin content remained below 20 fmol/cell at temperatures ranging from 12 to 27 °C. No toxins were detected at 25 psu for cells grown at 30 °C. At 12 and 18 °C, the proportion of C2 toxin decreased (from 100 to 7.3 mol%) when the salinity increased while GTX5 increased from 24.1 to 66.8 mol% (Table 1). 

GTX4 increased slightly with elevated salinity and C3 appeared at 30 psu for temperatures of 18, 21, 27 and 30 °C, thus showing values of 1.0 to 5.6 mol% toxin cell (Table 1). In the literature, the reported effects of salinity on toxin content are rather contradictory. For example, it was shown that an increase of salinity from 15 to 30–37.5 psu induced an increase in toxin production for *Alexandrium minutum* [11]. Conversely, other studies reported higher toxin content at low salinity, for example, in *Alexandrium tamarense* [5]. Grzebyk *et al.* (2003) [6] showed that the PST content of *A. minutum* developing in Brittany (France) was low (10 fmol/cell) from 30 to 37 psu, but it increased at lower salinities, with the maximum value (50 fmol/cell) recorded at 15 psu. However, in a salinity change experiment, where *Alexandrium fundyense* cells experienced short-term exposure to higher and lower salinities, with either acclimated growth at 38 psu or with short-term changes between 28 and 38 psu, no significant changes were observed in the toxin content of *A. fundyense* cells [10]. Lim and Ogata (2005) [7] examined the salinity effect on toxin production of four tropical *Alexandrium* species. Their results showed that the toxin content decreased with elevated salinities in *A. minutum*, the highest toxin content being about 12 fmol/cell at 5 psu. In *Alexandrium tamiyavanichii*, toxin content peaked at optimal growth salinity (20 and 25 psu). Toxin content of *A. tamarense*, somehow peaked at sub-optimal growth salinity (15 and 30 psu) [7]. These results suggest that the effects of salinity on *Alexandrium* species were not only region-dependent but also species-dependent. Our results showed that the observed variations (Figure 2) in total cellular toxin amount of *A. catenella* in response to salinity changes were related to changes in C2, GTX4 and GTX5 toxins. Hwang and Lu (2000) [11] showed that low salinity stimulated *A. minutum* cells to produce higher amounts of GTX1, but high salinity stimulated the cells to produce higher amounts of GTX2 and GTX3.

### 2.3. Influence of Temperature

It has been shown that temperature was the most important environmental factor driving seasonal variations of dinoflagellate abundances and growth in temperate coastal systems [2,8,38]. Low toxin amounts (<30 fmol/cell) were recorded for slowly growing ACT03 cells cultured at 12 °C (Figure 2). When the salinity of the culture medium ranged between 30 and 40 psu, the concentrations of toxins in *A. catenella* cells did not vary significantly with temperature between 18 and 30 °C. For cells grown in salinities between 10 and 25 psu there was no clear trend. However, toxins were not detected in cells growing at 30 °C. As compared to cell toxin contents measured at the highest salinities (30 to 40 psu), those determined at salinities ranging from 10 to 25 psu decreased substantially and varied from 4.37 ± 1.79 fmol/cell for 18 °C to 12.7 ± 5.1 fmol/cell for 12 °C. The toxin profile changed with temperature. At 12 °C, C2 dominated largely at almost all salinities except at 35 and 40 psu where C3 and C4 appeared but did not exceed 50 mol% of the total toxin amount. For temperatures higher than 18 °C, C2 toxins dominated at 10, 15 and 25 psu but not at 20 psu. The cells grown at 30, 35 and 40 psu showed an increasing concentration of C3 toxin and equal concentrations of C2 and C4 toxins. 

*Alexandrium* species have been shown to be more toxic at low temperature [9,10,18]. However, an isolate of *Alexandrium fundyense* from the Bay of Fundy (USA) displayed enhanced toxicity when grown at low (<10 °C) and high (25 °C) temperatures as compared to growth at 20 °C [8]. It was suggested that the increase in toxin content at low temperatures was not only due to low division rates but also associated with other factors, such as turnover rates of cellular components. Cells might allocate more cellular nitrogen to toxin synthesis and less to protein synthesis at low temperature. To compare these results to those obtained in the present study, we have to consider the wide temperature range (12 to 30 °C). Total toxin concentrations (fmol/cell) measured at 12 °C differ significantly (*P* < 0.05) from those measured at temperatures between 18 and 30 °C. However, *A. catenella* cells did not divide at 12 °C, showing less motile vegetative cells together with the formation of temporary cysts which suggested that physiological processes, including toxin biosynthesis and production, were negatively affected [38] at 12 °C. In Thau Lagoon, *Alexandrium* blooms develop at temperatures around 18–21 °C and salinities of 35 to 38 psu. Our results show that the total concentration of toxins did not change significantly (*P* < 0.05) for combined temperature and salinity values of 18–30 °C and 30–40 psu, respectively. The most noticeable changes occur for C2 and C4 toxin amounts which decreased and for GTX4 and GTX5 toxins, which substantially increased with temperature (Figure 2). We also show for the first time that there was a notable change in toxin content at the same temperature but with variable salinity values for both toxin content and profile (Figure 2, Table 1).

### 2.4. Influence of Irradiance on Toxin Content

**Figure 4:**
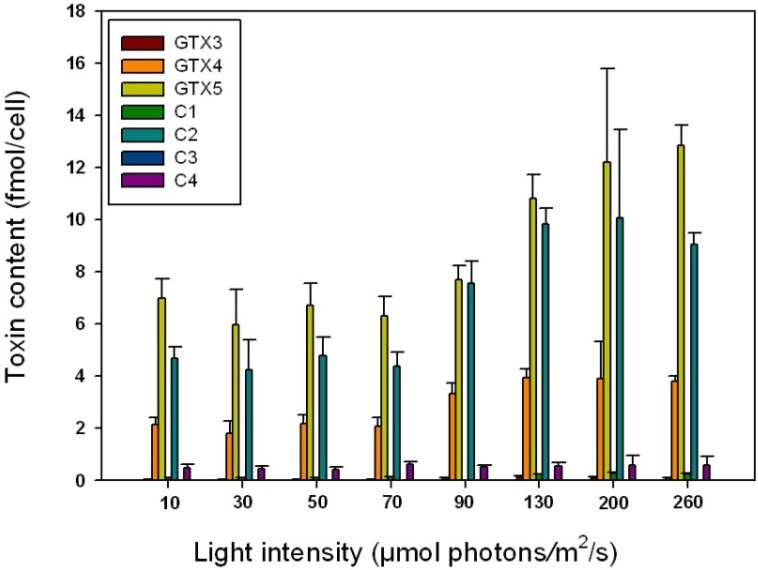
Toxin content (fmol/cell) of *Alexandrium catenella* as a function of irradiance. Cells were grown at a temperature of 20 °C and a salinity of 38 psu.

*Alexandrium catenella* cells started producing toxin at 10 μmol photons/m^2^/s, a very low irradiance level. The total toxin content of *A. catenella* was stable from 10 to 70 μmol photons/m^2^/s with a mean value of 13.7 ± 0.8 fmol/cell and then increased with irradiance (Figure 4). It reached a mean value of 24.7 ± 3.9 fmol/cell between 130 and 260 μmol photons/m^2^/s.

This trend was observed for all of the main toxins (GTX4, GTX5 and C2). The *N*-sulfocarbamoyl toxins C1, C4 and GTX3 did not show any clear variation in relation to light intensity (Table 2). The same tendency has been shown by Etheridge and Roesler (2005) [8] for *Alexandrium fundyense* isolates from the Gulf of Maine (MI isolate) and from the Bay of Fundy where toxicity increased with irradiance (from 6 to 175 μmol photons/m^2^/s). However, at the highest irradiance (425 μmol photons/m^2^/s) the MI isolate toxicity decreased drastically from 100 fmol/cell at standard conditions to 30 fmol/cell. These authors showed that although a relationship was observed between toxins and irradiance level, there was no direct relationship between the maximal photosynthetic rate and toxicity for either isolate during any of the experiments on *A. fundyense*.

**Table 2:** Molar percentage (mol%) of the toxins in *Alexandrium catenella* (strain ACT03) cells grown at different irradiances and at a temperature of 20 °C and a salinity of 38 psu.

Irradiance (μmol photons/m^2^/s)	GTX3	GTX4	GTX5	C1	C2	C4
10	1.3	65.2	12.7	-	19.9	0.9
30	2.1	62.1	14.8	-	20.2	0.7
50	1.4	61	15.8	-	20.6	0.9
70	1.6	61.1	17	-	19.5	0.8
90	0.6	62.9	16.9	-	18.4	1.2
130	1	61.2	16.9	-	19.5	1.4
200	1.1	63.4	16	-	18.5	1
260	0.7	63.4	16.4	-	17.9	1.6

### 2.5. Relationship between Growth and *Alexandrium catenella* Toxicity

**Figure 5:**
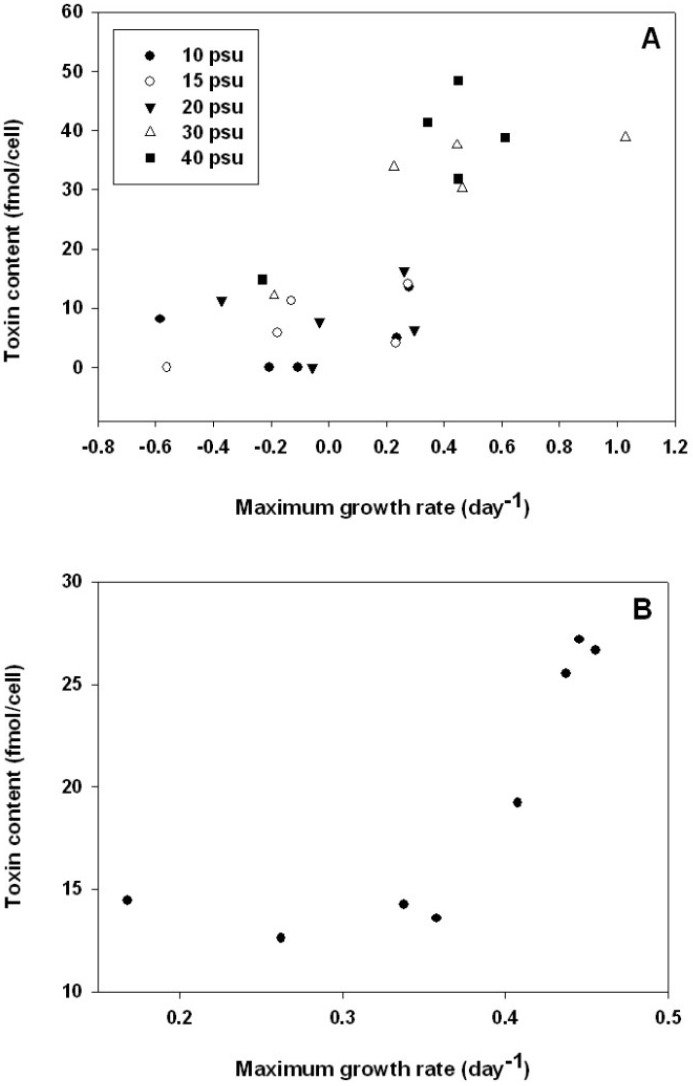
Relationship between maximum growth rate (day^−1^) and toxin content (fmol/cell) at (**A**) different combinations of temperatures (12, 18, 21, 24, 27 and 30 °C) and salinities (10, 15, 20, 30, 35 and 40 psu) and at (**B**) different irradiances (10, 30, 50, 70, 90, 130, 200, 260 μmol photons/m^2^/s); temperature was 20 °C and salinity was 38 psu.

For positive growth rates of *A. catenella* ranging between 0 and 1.1 day^−1^, a significant linear positive relationship was observed between toxin content and division rate (*R*^2^ = 0.86, *P* < 0.05, Figure 5A). In contrast, an inverse relationship was reported in other studies for *Alexandrium* species grown under various temperature and salinity conditions [9]. However, for *A. tamarense*, there was no correlation between growth rate and toxicity [39]. It has been suggested that environmental parameters could affect toxicity of HAB species by influencing the growth rate [40]. However the relationship between cellular toxicity and environmental factors revealed a complex relationship between growth rate and algal cell toxin profiles. In the present study, data on the cellular toxin content of *A. catenella* grown at different irradiances revealed a significant positive relationship between growth rate and cell toxin content (*R*^2^ = 0.63; *P* < 0.05, Figure 5B). It has been suggested that the toxicity of *A. fundyense* was not directly related to physiological responses such as photosynthetic and growth rates and it was concluded that toxicity cannot be modeled solely as a function of growth rate [8]. To address these contrasting results, it is necessary to study the relationship between growth rate and toxicity in different environmental conditions for each cultured strain.

## 3. Experimental Section

### 3.1. Culture of *A. catenella*

**Figure 6:**
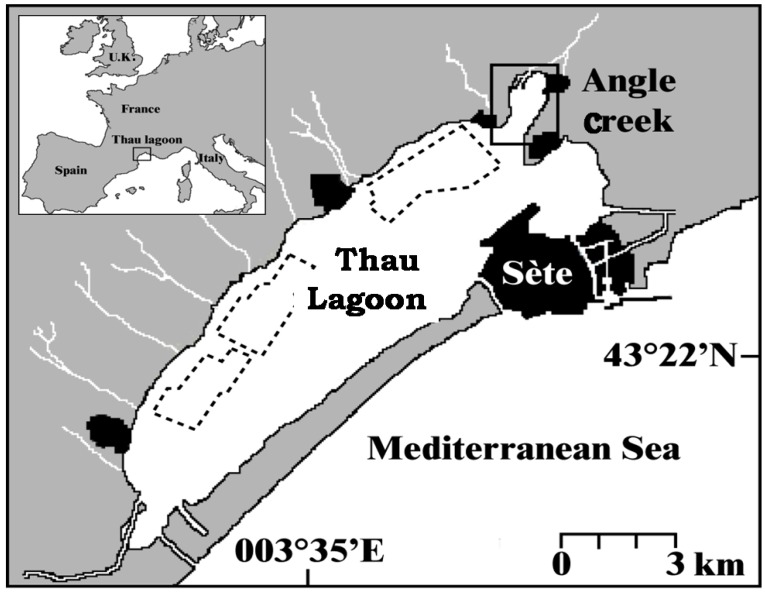
Thau Lagoon and the Angle Creek where *Alexandrium* blooms develop.

The *A. catenella* strain ACT03 used in this study was obtained after isolation of a single vegetative cell from a seawater sample that was collected during a toxic bloom event in October 2003 in Thau (Figure 6). According to its rDNA sequence, this strain belongs to Group IV of the *A. tamarense* species complex [21,41]. Since then, ACT03 has been grown and kept in batch cultures on the sterilized artificial medium ESAW (Enriched Seawater Artificial Water), without silicates [42] at 38 psu, at a temperature of 20 °C and under a cool-white fluorescent illumination (100 μmol photons/m^2^/s) on a 12 h:12 h light:dark cycle. The experiments were carried out using sterile flasks, each containing 30 mL of culture medium inoculated with 200 cells/mL. All the experiments were carried out in triplicate cultures.

The toxicity of the cells was studied using a crossed experimental design with 42 different combinations, obtained from combining seven temperatures (12, 18, 21, 24, 27 and 30 °C) and six salinities (10, 15, 20, 25, 30, 35 and 40 psu) under an irradiance of 100 μmol photons/m^2^/s. To prevent any temperature or salinity shock, the cultures were pre-acclimated to the desired experimental conditions via stepwise transfer over a period of ≥25 days [43,44].

For the experiments designed to investigate the effect of irradiance, the inoculum culture was grown at 20 °C and 100 μmol photons/m^2^/s and the salinity was set at 38 psu. Eight light intensities were then tested: 10, 30, 50, 70, 90, 130, 200 and 260 μmol photons/m^2^/s. These intensities were obtained by attenuating the cool-white fluorescent light with absorbing vinyl screens and further controlled using a quantum light meter (LI-COR Quantum/Photometer).

For both experiments, every day for two weeks, the experimental flasks were gently shaken and 500 μL samples were fixed using Lugol’s iodine solution. Cell concentration was monitored daily via direct microscopic counts using a Nageotte counting chamber. In accordance with Guillard (1973) [45], the specific growth rate (μ; expressed in day^−1^) was calculated from the slope of a linear regression over the entire exponential phase of growth by the least square fit of a straight line to the data after logarithmic transformation; μ = (Ln *N*_t_ − Ln *N*_0_)/(*t*_1_ − *t*_0_) in units of day^−1^ where *N*_0_ and *N*_t_ represent the cell density in cells/mL at the start, *t*_0_, and end, *t*_1_, of the exponential phase, respectively. Growth was monitored until the maximum cell concentration was reached.

### 3.2. Toxin Quantification by Liquid Chromatography/Fluorescence Detection

The toxin content and composition of *A. catenella* cells were determined in the late exponential phase of growth. The triplicates of 30 mL batch cultures were harvested during the exponential growth phases when the cell concentration was ≥3 × 10^3^ cells/mL. After centrifugation (3000× *g*, 8 min, 4 °C), the cells were suspended in 1 mL of 0.1 N acetic acid and frozen at −20 °C until the extraction and toxin analyses were performed. To release toxins, the samples were sonicated for 5 min in a water bath three times, and centrifuged at 17,000× *g* for 10 min at 4 °C. The supernatants were used for the subsequent LC/FD PSP toxin analyses, using the method of Oshima (1995b) [46] with slight modifications. Toxins were separated by reverse chromatography using a C8 column (5 μm Develosil, 4.6 mm i.d. × 250 mm) with a flow rate of 0.8 mL/min. Toxins were quantified using certified standards provided by the CNRC (Halifax, Canada). C-toxins (C1–4) were detected and quantified indirectly after acid hydrolysis (HC 0.4 N at 97 °C for 5 min) [26]. The acid hydrolysis transformed the C1, C2, C3, C4 toxins into GTX2, GTX3, GTX1, GTX4, respectively.

### 3.3. Statistics

Data were analyzed using one-way ANOVA (Statistica 10.0 software). For correlations between variables, linear regressions were performed using Sigmaplot/statistics Fit Curve application, *R*^2^ and *P* value are indicated.

Values are means of triplicate cultures at each condition.

## 4. Conclusion

Toxic *Alexandrium* species occur worldwide and are able to produce various paralytic shellfish toxins. The most abundant toxins in *Alexandrium catenella* strains developing in Mediterranean, Asiatic and South American waters are C1,2 and GTX1,2,3,4, whereas STX and NeoSTX are almost absent (Table 3 and references therein). *Alexandrium tamarense* develops in large geographic regions and shows a toxin profile close to that of *A. catenella* strains with C1,2 and GTX1,2,3,4 dominating the other toxins (Table 3). For *Alexandrium minutum* colonizing Mediterranean, Asiatic and European Atlantic waters, GTX1,2,3,4 dominate the other toxins. This has been mentioned by Cembella *et al*. (1987) [28] for members of the *A. minutum* group. In contrast, *Pyrodinium bahamense*, a PST producer, shows high percentages of STX and NeoSTX [47]. For an *Alexandrium fundyense* strain isolated from Casco Bay (Maine, USA) GTX3,4; NeoSTX; STX and C2 were the major toxins [15]. Nevertheless, for numerous species of the cosmopolitan *Alexandrium* genera investigated worldwide, STX and NeoSTX were generally reported as traces or found in low concentrations (Table 3). The ACT03 toxin profile and data gathered in Table 3 are consistent with reports of Anderson *et al.* (2012) [2] showing that in *Alexandrium* species, decarbamoyl derivatives (dcSTX, dcNEO dcGTX1-4) and the *N*-21 sulfocarbamoyl analogs C3 and C4 are rarely found. Table 3 shows that a non-negligible diversity in toxin profiles occurred among natural populations as well as in cultured species or clones of the *Alexandrium tamarense* complex which is not in favor of using PST as a reliable genetic fingerprint for chemotaxonomic characterization.

**Table 3:** Summary of the toxin composition and cellular toxin content (fmol/cell) for worldwide distributed dinoflagellate species and strains producing PST toxins. Salinity (psu), temperature (°C) and irradiance (μmol photons/m^2^/s) conditions for the cultures are reported when available.

Species and studied area	Temp	Sal	Irradiance	Toxins	Toxin content	Reference
	(°C)	(psu)	(μmol/m^2^/s)	(molar basis in %)	(fmol/cell)	
***Mediterranean waters***						
*Alexandrium catenella* ATTL01,02 (Thau, France)	15	31	200	**PT**: C1,2 > GTX1,4 > GTX2,3	5.3–44.3 fgEqSTX/cell	Lilly *et al.* (2002) [21]
*Alexandrium catenella* ACT03 (Thau, France)	21–27	35–40	90–130	**PT**: GX5 > C2 > C4 > GTX4 > GTX3 ≈ C1 ≈ C3	2.9–50.3	This study
*Alexandrium minutum* (Greek coastal waters)	20	37.5	60	**PT**: GTX1; GTX4	-	Ignatiades *et al.* (2007) [29]
*Alexandrium minutum* (Spain*)*	17.5	35	-	**PT**: GTX4 > GTX1 > GTX3; GTX2	0.1–0.4	Dacosta *et al.* (2008) [31]
***Asian waters***						
*Alexandrium catenella* (Japan)	15	50	*-*	**PT**: C2 (70.3%); NeoSTX(19.9%); C1 ≈ GTX1 ≈ STX	34.5 ± 23	Sekiguchi *et al.* (2001) [48]
*Alexandrium catenella* (Hong Kong)	20–23	33	-	**PT**: GTX4 > GTX3 > GTX1 > GTX6 > Neo STX; Cx	-	Siu *et al.* (1997) [36]
*Alexandrium catenella* (China)	23.5	-	120	**PT**: C1,2 (80%–90%) and GTX1,4 (5%–15%)	-	Xu *et al.* (2012) [35]
*Alexandrium catenella* (East China Sea)	20	-	80	**PT**: C1,2 (predominant)/trace amounts of GTX1,3,4,5,6	4–14	Li *et al.* (2011) [34]
*Alexandrium tamarense* (Japan)	15	-	-	**PT**: C1,2 > GTX1,4 > GTX2,3/traces of NeoSTX and STX	-	Ichimi *et al.* (2002) [49]
*Alexandrium tamarense* (ATHK01, Hong Kong)	22	-	220	**PT**: GTX1,4; GTX2,3; STX	1.64	He *et al.* (2010) [50]
*Alexandrium tamarense* HK9301 (Hong Kong)	23^+^	25–30	80–220	**PT**: C1,2 > GTX4 > GTX5 > GTX1 > C3,4	15–85	Wang and Hsieh (2005) [14]
*Alexandrium tamarense* (Southeast China sea)	12–24	15–30	60	**PT**: C1,2 (60%–80%); GTX5 (15%–30%); traces of GTX1,3,4	8–55	Wang *et al.* (2006) [51]
*Alexandrium tamarense* AtPA01 (Malaysia)	25^+^	10–30	140^+^	**PT**: GTX1,4 (25%–40%); NeoSTX (30%–40%)	0.1–0.8	Lim and Ogata (2005) [7]
*Alexandrium peruvianum* ApKS01 (Malaysia)	25^+^	10–30	140^+^	**PT**: GTX1,4,5,6	0.25–0.75	Lim and Ogata (2005) [7]
*Alexandrium minutum* (Malaysia)	25^+^	10–30	140^+^	**PT**: GTX1,4 (95%); MT: GTX2,3; traces of STX; NeoSTX	0.27–2.08	Lim and Ogata (2005) [7]
*Alexandrium minutum* T1 (Taiwan)	25	15	120	**PT**: GTX1,4 > GT2,3	-	Hwang and Lu (2001) [11]
*Alexandrium minutum* (Taiwan)	20–22	-	60	**PT**: GTX1,4 or GTX2,3 depending on the strains	-	Chou *et al.* (2004) [52]
*Alexandrium affine* (Vietnam)	24	30	25	**PT**: GTX1,4; NeoSTX; STX	1–2.28	Nguyen-Ngoc (2004) [53]
*Alexandrium tamiyavanichi* (Japan)	25	30	100	**PT**: C2; GTX4/**MT**: C3,4; GTX2,3,5; STX; NeoSTX	-	Oh *et al.* (2009) [54]
*Gymnodinium catenatum* (Australia)	-	-	-	**PT**: C1,2; C3,4; B1,2; NeoSTX	-	Hallegraeff *et al.* (2012) [55]
*Pyrodinium bahamense* (Philippines)	-	-	-	**PT**: STX (85%–98%)/**MT**: dcSTX; B1	54–298	Gedaria *et al.* (2007) [47]
***North American Atlantic waters***						
*Alexandrium fundyense* (Casco Bay, Maine)	15	-	200	**PT**: GTX3,4; NeoSTX; STX ; C2 MT: GTX1,2,5; dcGTX3; C1	≈141	Poulton *et al.* (2005) [15]
*Alexandrium fundyense* MI (Gulf of Maine, USA)	20	30	100	**PT**: GTX2,3; GTX1,4 > STX > NeoSTX	244	Etheridge and Roesler (2005) [8]
*Alexandrium tamarense* Pr18b (Canada)	10–30	25	40–470	**PT**: C1,2 (64.0%), GTX1,2,3,4 (1.7%); STX-NeoSTX (16.2%–17.8%)	140–450	Parkhill and Cembella (1999) [20]
***South American waters***						
*Alexandrium catenella* (South of Chile)	12–14	30	50	**PT**: C1,2; GTX1,4/scarcity of absence of STX	41.4–295.5	Varela *et al.* (2012) [37]
*Alexandrium catenella* (South of Chile)	15	-	50	**PT**: GTX1,4 > GTX2,3, (they represent more than 98%)	-	Aguilera-Belmonte *et al.* (2011) [56]
*Alexandrium tamarense* (Argentina, Brazil, Chile)	-	-	-	**PT**: C1,2 (predominant); GTX1,4/**MT**: STX	17–261	Montoya *et al.* (2010) [57]
*Alexandrium tamarense* (Southern Brazil)	20	-	250	**PT**: C1,2 (30%–84%); GTX1,4 (6.6%–55%); GTX2,3 (<29%); NeoSTX (<24%)	-	Persich *et al.* (2006) [58]
***European Atlantic waters***						
*Alexandrium tamarense* (Coast of Greenland)	10	-	30	**PT**: GTX1,2,3,4	-	Baggesen *et al.* (2012) [59]
*Alexandrium minutum* (Irish coastal waters)	15	-	-	**PT**: GTX3 (>80%) and GTX2	14 pg/cell	Touzet *et al.* (2007) [60]
*Alexandrium minutum* AM89BM (France)	18	25	100	**PT**: GTX2,3 (>97%)	≈30.4	Grzebyk *et al.* (2003) [6]
*Alexandrium minutum* (Fleet Lagoon, UK)	17	-	120	**PT**: GTX3 (54%); GTX2 (10%); STX (36%)	5.6–16.8	Nascimento *et al.* (2005) [61]

(PT) Principal Toxins, (MT) Minor Toxins, (-) data not available. Cellular toxin content is expressed in fmol/cell or other specified unit.

Regarding the cellular toxin content, it is notable that a wide range of variation (0.1 to 450 fmol/cell) occurred among (or even within) *Alexandrium* species occupying different geographical locations with contrasted environmental conditions and when considering cultured or natural populations (Table 3 and references therein). This clearly signifies that the cell PST content cannot be used as a species-clone marker. We showed that the changes in total cellular toxicity of *A. catenella* observed in response to the environmental conditions were due to both changes in the overall concentrations of toxins and in the relative toxin composition (Figure 2, Figure 3). Results suggest that it is important to consider the combined influence of environmental parameters on HAB species toxicity as responses may differ when microalgae are faced with changes in only one abiotic factor. In the present study, unlike the toxin content per cell which varies notably when *A. catenella* grows at different temperature, salinity, and light conditions, the toxin profile remains almost constant. However, we observe significant changes in the toxin profile of this dinoflagellate at salinities of ≤25 psu or when the temperature was 12 °C. It is important to underline that these environmental conditions did not prevail during *Alexandrium* bloom periods in Thau. Our results are in agreement with previous studies [10,62,63] showing that cellular toxin content is a less stable phenotypic character among and within *Alexandrium* species than the relative composition of PSP toxins, which changes significantly only under extreme growth conditions. Laboratory studies on the effect of different combinations of temperature, salinity and light on the growth and toxicity of *A. catenella* together with *in situ* investigations on bloom dynamics of this dinoflagellate can help us improve our monitoring and modeling of toxic events both spatially and temporally.
